# Antibody Therapy of Pediatric B-Cell Lymphoma

**DOI:** 10.3389/fonc.2013.00068

**Published:** 2013-04-02

**Authors:** Friederike Meyer-Wentrup, Verena de Zwart, Marc Bierings

**Affiliations:** ^1^Department of Hematology and Oncology, Wilhelmina Children’s Hospital, University Medical Center UtrechtUtrecht, Netherlands

**Keywords:** pediatric B-cell lymphoma, therapeutic antibodies, cancer therapeutics, individualized medicine, cancer immunotherapy

## Abstract

B-cell lymphoma in children accounts for about 10% of all pediatric malignancies. Chemotherapy has been very successful leading to an over-all 5-year survival between 80 and 90% depending on lymphoma type and extent of disease. Therapeutic toxicity remains high calling for better targeted and thus less toxic therapies. Therapeutic antibodies have become a standard element of B-cell lymphoma therapy in adults. Clinical experience in pediatric lymphoma patients is still very limited. This review outlines the rationale for antibody treatment of B-cell lymphomas in children and describes potential target structures on B-cell lymphoma cells. It summarizes the clinical experience of antibody therapy of B-cell lymphoma in children and gives an outlook on new developments and challenges for antibody therapy of pediatric B-cell lymphoma.

## Rationale for Antibody Therapy of B-Cell Lymphomas in Children

Pediatric lymphomas account for 10% of all pediatric malignancies (Howlader et al., 2009). Approximately 60% are of B-cell origin. They comprise Hodgkin and B-Non-Hodgkin lymphomas (HL and B-NHL). Among B-NHL Burkitt lymphomas are the most frequent, followed by diffuse large B-cell lymphomas. Current B-cell lymphoma treatment in children consists of four modalities: chemotherapy, surgery, radiotherapy, and in some cases allogeneic stem cell transplantation (Reiter, 2007; Carbone et al., 2011; Miles et al., 2012; Shankar and Daw, 2012). With current treatment protocols, overall 5-year event-free survival of pediatric Hodgkin and B-Non-Hodgkin lymphoma patients is between 80 and 90% (Reiter, 2007; Carbone et al., 2011; Miles et al., 2012; Shankar and Daw, 2012). In view of this high cure rate, current treatment protocols focus on reducing chemotherapy intensity without decreasing overall and event-free survival. However, a relevant further reduction of chemotherapy intensity with current chemotherapeutics is not very likely. In children late effects of chemotherapy are particularly important, calling for development of new therapeutic options with reduced toxicity. Since chemotherapeutic drugs target not only tumor but also healthy cells, they have a number of side effects. Mainly rapidly dividing cells of the body are affected resulting in significant toxicity such as cytopenia (leading to increased bleeding tendency and increased risk of infections), gastrointestinal mucositis, cardiac, kidney or liver toxicity, reduced fertility, and alopecia. Treatment may even be postponed because of critically low immune cells or infectious complications. An important long term side effect is the increased risk of secondary malignancies. Allogeneic stem cell transplantation can be accompanied by severe acute and chronic toxicity (infections, non-engraftment, acute, and chronic graft versus host disease) resulting in treatment-related mortality of approximately 10–20%, depending on comorbidities and best available donor-match (Satwani et al., 2012). Limited therapeutic options for relapsed disease are another major treatment challenge often leading to poor prognosis. Hence, there is a medical need for new, better targeted therapies. One form of targeted therapy that over the past decade has been increasingly recognized as being effective in cancer treatment is monoclonal antibody therapy (Weiner et al., 2010; Scott et al., 2012). Monoclonal antibodies contain uniform variable regions and are thus specific for a single epitope (Kohler and Milstein, 1975, 2005). Directed against a tumor-specific antigen, these antibodies can target and destroy tumor cells, ideally without harming healthy tissue. To reduce antibody inactivation by the human immune system originally murine monoclonal antibodies can be genetically modified to resemble the human counterpart (humanized or chimeric antibodies). Monoclonal antibodies can be produced in pharmaceutical grade using recombinant DNA technology (Chon and Zarbis-Papastoitsis, 2011). The optimal B-cell lymphoma target antigen should be expressed on the cell surface, so it can easily be reached by the antibody. It should be uniquely or preferentially (over-) expressed by malignant B-cells. There should be little or no expression by B-cell precursors enabling reversible B-cell depletion and fast repopulation after therapy. Even after antibody binding expression of the target antigen should remain high (it should not shed or internalize in response to antibody binding). Finally, the antigen should be essential for B-cell lymphoma cell survival or homeostasis to reduce the likelihood of antigen loss resulting in lymphoma immune escape variants. In case of nodular sclerosis classical Hodgkin lymphoma antibodies targeting cells of the tumor microenvironment can also be a therapeutic option. This increases the number of potential targets and applicable anti-lymphoma antibodies.

The exact working mechanism for antibody-mediated lymphoma cell destruction may vary between different therapeutic antibodies. In general, antibody-dependent cell-mediated and complement-dependent cytotoxicity, phagocytosis by Fc-receptor-expressing immune cells, antigen cross-presentation, and apoptosis induction are all thought to contribute to the effectiveness of antibody treatment (Weiner et al., 2010). Side effects of reversible B-cell depletion are considered to be tolerable, especially since immunoglobulins can be substituted to prevent infections.

## Available Therapeutic Antibodies Targeting Pediatric B-Cell Lymphomas

Truly B-cell lymphoma specific targets have not yet been identified. All available therapeutic antibodies target proteins that are also expressed by normal B-cells. Expression may be universal (e.g., CD19) or restricted to B-cell subpopulations (e.g., CD30). Possible side effects of lymphoma antibody therapy are therefore a direct consequence of target protein expression by normal B-cells. B-cell lymphoma surface molecules that at least partly fulfill the above mentioned requirements for antibody targets are CD19, CD20, CD21, CD22, and CD30 (see Table 1 for an overview).

**Table 1:** **Putative antibodies for B-cell lymphoma therapy in children**.

Targeted antigen	Therapeutic antibody	Published results for pediatric lymphoma patients (references/FDA phase study performed)	Trials open for pediatric lymphoma patients (FDA phase, clinicaltrials.gov Identifier)	Clinical experience in other pediatric diseases	FDA-approved for adults
**MONOSPECIFIC**					
CD19	SAR3419 (drug-conjugate)	No	No	Yes, phase 2 study open for leukemia patients ≥16 years (NCT01440179)	No
	SGN-CD19A (drug-conjugate)	No	Yes, phase 1 (NCT01786135)	No	No
CD20	Rituximab	Yes (Meinhardt et al., 2010; Goldman et al., 2012), phase 1/2 studies	Yes	Yes	Yes (lymphoma)
				Immune thrombocytopenic purpura (Bennett et al., 2006; Patel et al., 2012)	
			Phase 3 (NCT01516580)	Autoimmune diseases (Jansson et al., 2011)	
			Phase 2/3 NCT01595048	Severe refractory juvenile idiopathic arthritis (Alexeeva et al., 2011)	
			Phase 2 NCT01700946	Steroid- and calcineurin-dependent nephritic syndrome (Ravani et al., 2011)	
			Phase 2 NCT00001337	Immunosuppression related lymphoproliferative disease (Messahel et al., 2006)	
				Systemic lupus erythematosus (Willems et al., 2006)	
			Phase 2 NCT00654732	Juvenile dermatomyositis (Oddis et al., 2013)	
			Phase 2 NCT01009970	Post-transplant lymphoproliferative disorder (Gupta et al., 2010)	
	Tositumomab and ^131^I-tositumomab (drug-conjugate)	No	No	No	Yes (lymphoma)
	^90^Y-Ibritumomab tiuxetan (drug-conjugate)	Yes (Cooney-Qualter et al., 2007)	No	No	Yes (lymphoma)
	Obinutuzumab	No	No	No	No
CD21	131I-OKB7	No	No	No	No
CD22	Inotuzumab Ozogamicin (drug-conjugate)	No	No	No	No
	Epratuzumab	No	No	No	No
	Moxetumomab pasudotox (drug-conjugate)	No	Yes, phase 1 (NCT00659425)	No	No
CD30	Brentuximab vedotin (drug-conjugate)	No, phase 1 study performed	Yes, phase 1/2 (NCT01421667) (NCT01492088)	No	Yes (lymphoma)
CTLA-4	Ipilimumab	No	Yes, phase 1 (NCT01445379)	No	Yes (melanoma)
PD-1	BMS-936558	No	No	No	No
**BISPECIFIC**					
CD19/CD3	Blinatumomab	No	No	Yes, phase 1 and 2 study open for leukemia patients (NCT01471782)	No

### CD19

CD19 forms the B-cell co-receptor together with CD21 and CD81. Anti-CD19 antibodies have *in vitro* and *in vivo* (mouse models) activity against CD19-expressing lymphomas (Horton et al., 2008; Ward et al., 2011). A CD19 antibody-drug conjugate (SAR3419) has recently been tested in a phase I trial in adult lymphoma patients (Younes et al., 2012a). Six of 35 patients achieved partial or complete remissions. CD19 is expressed by most B-cell lymphomas and all normal B-cells. A bispecific anti-CD19/anti-CD3 antibody (Blinatumomab) has shown good clinical results in adult lymphoma patients alone (Bargou et al., 2008) and in combination with the anti-CD20 antibody Rituximab (d’Argouges et al., 2009). When given alone to relapsed adult NHL patients Blinatumomab induced four complete and seven partial tumor regressions among 38 patients treated. These effects were dose-dependent (Bargou et al., 2008). Handgretinger et al. (2011) report induction of complete remission in three children with post-stem cell transplantation acute lymphocytic leukemia relapse after treatment with Blinatumomab. CD19 can form heterodimers with CD21. High CD21 expression on lymphomas has been shown to inhibit internalization of anti-CD19 antibodies into lymphoma cells leading to reduced efficacy of anti-CD19-drug conjugates (Ingle et al., 2008). However, recently Gerber et al. (2009) reported activity of an anti-CD19-auristatin-conjugate antibody (hBU12-vcMMAE) against lymphoma cells even in the presence of high CD21 expression.

### CD20

CD20 is expressed by almost all pediatric B-NHL (Lones et al., 2000; Swerdlow et al., 2008) and by normal B-cells. Its precise cellular role remains to be elucidated. CD20 induces transmembrane Calcium flux, either directly by functioning as a Calcium channel or indirectly by modifying the function of other Calcium channels (Bubien et al., 1993; Li et al., 2003; Walshe et al., 2008).

Most clinical experience exists for the anti-CD20 antibody Rituximab. It was FDA-approved for the treatment of relapsed or refractory low-grade or follicular B-NHL in adults in 1997. Anti-CD20 treatment leads to profound B-cell depletion. Rituximab is used successfully for treatment of adult B-cell lymphomas and B-cell-mediated autoimmune diseases. Pharmacokinetics and -dynamics of Rituximab have been studied in adult patients (McDonald et al., 2010; Tran et al., 2010; Jäger et al., 2012). Pediatric pharmacokinetic and -dynamic data are very limited (Pranzatelli et al., 2010). Consequently, the adult dose is usually given also to children, adjusted for body surface. A radiolabeled anti-CD20 antibody (90yttrium-ibrituxomab-tiuxetan) has been tested in a phase I study in children with relapsed and refractory CD20-positive NHL (Cooney-Qualter et al., 2007). Patients did not experience dose-limiting toxicity. The authors have designed a phase II study to further evaluate safety and clinical efficacy of radiolabeled anti-CD20 antibody.

### CD21

CD21 is complement receptor-2 and forms part of the B-cell co-receptor complex. It is expressed by mature B-cells, some B-cell lymphomas, and thymocytes, a subset of peripheral T-lymphocytes (Fischer et al., 1999). The anti-CD21 antibody-131I-conjugate 131I-OKB7 was evaluated in a phase I study in 18 adult lymphoma patients. Antitumor responses to a varying degree were observed in 13 of 18 patients (Czuczman et al., 1993). However, phase II study results have not been published.

### CD22

CD22 is a sialic acid-binding immunoglobulin-like lectin (SIGLEC) and functions as an immune inhibitory receptor on B-cells (Poe and Tedder, 2012). It is expressed by all mature B-cell populations and some B-cell lymphomas. CD22 is endocytosed upon ligand binding. Therapeutic monoclonal anti-CD22 antibodies conjugated to a cytotoxic agent (Inotuzumab ozogamicin, CMC-544) have shown anti-lymphoma efficacy in a phase I study in adult B-NHL patients (Advani et al., 2010). Treatment data for pediatric lymphoma patients have not been published. Recently, de Vries et al. (2012) described efficient killing of primary pediatric B-cell precursor ALL cells by CMC-544. Epratuzumab, another anti-CD22 antibody, has shown clinical efficacy in the reinduction therapy in children with All in bone marrow relapse (Raetz et al., 2008) and in combination with Rituximab and chemotherapy in the treatment of adults with diffuse large B-cell lymphoma (Micallef et al., 2011).

### CD30

The T- and B-lymphocyte activation antigen CD30 is expressed by mononuclear Hodgkin cells and multinucleated Reed-Sternberg cells (Swerdlow et al., 2008). CD30 is a member of the tumor necrosis factor receptor superfamily. An anti-CD30 antibody (SGN-30) showed antitumor activity *in vitro* and in Hodgkin lymphoma animal models (Wahl et al., 2002). The clinical effects in lymphoma patients, however, were disappointing (Wahl et al., 2002; Bartlett et al., 2008; Forero-Torres et al., 2009). Only when it was conjugated to a cytotoxic drug (monomethyl auristatin E) anti-Hodgkin lymphoma activity was observed in patients (Younes et al., 2010). The anti-CD30 antibody-drug conjugate Brentuximab vedotin was FDA-approved in 2011 for the treatment of adult patients with Hodgkin lymphoma after failure of autologous stem cell transplantation or after failure of at least two prior multi-agent chemotherapy regimens in patients who are not candidates for hematopoietic stem cell transplantation (Younes et al., 2010, 2012b). Currently, two phase 1 studies are recruiting pediatric Hodgkin lymphoma patients (Table 1).

## Current Clinical Experience with Antibody Therapy of B-Cell Lymphomas in Children

The largest body of clinical data exists for Rituximab (anti-CD20). In adults, Rituximab has been applied successfully to treat B-cell lymphomas (Coiffier et al., 2002; Pfreundschuh et al., 2006, 2008) and has become part of standard therapy. Pediatric randomized controlled trials on antibody B-cell lymphoma therapy have not been published. The published clinical data consist of case reports, case series, and phase I and II studies. Meinhardt et al. (2010) report a response rate of 44% in a phase II window study of Rituximab treatment of 87 children diagnosed with mature B-NHL and Burkitt leukemia. Patients received the adult dose of 375 mg/m^2^ Rituximab during 5 days prior to starting chemotherapy. Kumar et al. describe treatment results of six children with follicular lymphoma treated with Rituximab (Goldman et al., 2012) and multi-agent chemotherapy. Five of six treated patients were in remission with a median follow-up of 31 months (Kumar et al., 2011). Griffin et al. (2009) report combining Rituximab with chemotherapy for six patients with diffuse large B-cell lymphoma and 14 patients with Burkitt lymphoma and B-ALL. The overall response rate (complete and partial) was 60% with acceptable toxicity. Finally, Goldman et al. (2012) recently reported treatment results of 45 children and adolescents with stage III/IV B-cell NHL treated with a combination FAB/LMB 96 chemotherapy and Rituximab. The 3-year event-free and overall survival was 95%. Currently, one phase 2/3, four phase 2, and one phase 1 study are open for pediatric B-cell lymphoma patients (Table 1). Side effects of Rituximab therapy in pediatric lymphoma patients are rarely reported (Meinhardt et al., 2010; Goldman et al., 2012). It remains to be determined if this is due to low incidence of side effects or low patient numbers evaluated so far.

## New Concepts for B-Cell Lymphoma Antibody Immunotherapy

While even the role of Rituximab in the treatment of pediatric B-cell lymphomas still has to be evaluated, new concepts for antibody lymphoma immunotherapy are already evolving. The most promising concepts focus on improving cytotoxicity of tumor-specific antibodies, on targeting immune modulatory molecules and the application of antibody combinations (Beck et al., 2010; Weiner et al., 2010).

There are different ways to increase antibody cytotoxicity. One of them is generation of bispecific antibodies. These antibodies are directed against two target antigens, e.g., a tumor antigen, such as CD19, and a molecule expressed on cytotoxic T-cells, such as CD3. CD19/CD3-bispecific antibodies bring lymphoma cells in close vicinity to cytotoxic T-cells thereby increasing killing of lymphoma cells (Loffler et al., 2000). Blinatumomab, a bispecific antibody targeting CD3 and CD19 has induced tumor regression in adult B-cell NHL patients (Bargou et al., 2008). Bispecific antibodies can also be directed against two antigens both expressed by lymphoma cells. Recently, Gupta et al. (2012) reported inducing lymphoma cell death with a bispecific antibody that crosslinks the B-cell antigens CD20 and CD74. Antibody glycoengineering is another important technique to increase cytotoxicity via improved Fc-receptor binding. This technique not only increases antibody-dependent cellular cytotoxicity, but also Fc-receptor mediated phagocytosis and antigen presentation. Obintuzumab (Braza et al., 2011), a glycoengineered anti-CD20 antibody has been tested in phase I studies in adult lymphoma patients (Salles et al., 2012; Sehn et al., 2012). In both studies heavily pretreated B-NHL patients received the antibody. Sehn et al. report five patients (23%) with partial response and 12 (54%) with stable disease at the end of the induction period. Salles et al. describe five complete and four partial responses (24 and 17%, respectively). A glycoengineered anti-CD19 antibody (MEDI-551) has shown increased cellular cytotoxicity *in vitro* and in murine animal models. The combination with Rituximab resulted in prolonged suppression of lymphoma growth (Ward et al., 2011).

Coupling of antibodies to cytotoxic agents (radionuclides, immunotoxins) is a third way to increase clinical efficacy. Brentuximab vedotin is a good example for this concept (Younes et al., 2012b). The unconjugated antibody did not induce clinical responses. Coupling of the same antibody to the cytotoxic agent monomethyl auristatin E resulted in a therapeutic agent that has received FDA approval for treatment of Hodgkin lymphoma in 2011.

Recently, antibodies targeting immune regulatory molecules such as anti-CTLA-4 (Ipilimumab) (Ansell et al., 2009) and anti-PD-1 (Norde et al., 2012; Pardoll, 2012; Topalian et al., 2012) have shown clinical efficacy as anti-cancer treatment. In a phase I study Ipilimumab induced a clinical response in 2 of 18 treated adult B-NHL patients (one ongoing complete response >31 months and a partial response lasting 19 months) (Ansell et al., 2009). PD-1 ligand expression on primary Hodgkin/Reed-Sternberg cells and PD-1 expression on HL-infiltrating T-cells has recently been demonstrated. Blockade of PD-1 signaling restored IFNγ production by lymphoma-infiltrating T-cells (Yamamoto et al., 2008). Anti-PD-1 antibodies are currently evaluated as treatment for HL and B-NHL in a phase I trial (NCT01592370).

Finally, combinational antibody therapy, e.g., combining antibodies against two or more lymphoma antigens (Alinari et al., 2011; Micallef et al., 2011; Ogura et al., 2012) or against a lymphoma antigen and an immune regulatory molecule, may further improve therapeutic efficacy. Therapeutic antibodies are usually given in combination with chemotherapy. Optimizing this chemoimmunotherapy to select the best antibody/chemotherapy combinations may also increase therapeutic efficacy (Harrold et al., 2012).

## Ways to Increase Participation of Children in New Developments of Antibody B-Cell Lymphoma Therapy

Experience with use of therapeutic antibodies in adult lymphoma patients is rapidly expanding. The successes of antibodies such as Rituximab and Ipilimumab have sparked the development of biosimilars (Carey, 2011), and of antibodies targeting other lymphoma antigens or immune regulatory molecules. These developments increase therapeutic options for lymphoma patients. Low patient numbers, however, make efficient clinical testing of new antibody-based therapies for children in randomized controlled trials in a timely fashion very difficult. This excludes children from profiting from new therapeutic developments in an evidence-based manner. At the same time extrapolation of adult data to pediatric patients is problematic, since tumor and patient physiology differ significantly (Murphy, 1980; Deffenbacher et al., 2012). Especially for high risk or relapsed patients this situation very much limits therapeutic options and may even prevent any further therapy. However, children with rare, potentially fatal diseases should be able to participate in new therapies as fast as possible.

National and international registries of all pediatric B-cell lymphoma patients receiving antibody-based therapies would be a first step to increase available treatment data. In addition, antibody-therapy must be included and evaluated in future national and international cooperative B-cell lymphoma therapy trials. It is encouraging that currently at last six studies evaluating Rituximab in pediatric B-cell lymphoma treatment are recruiting patients (see Table 1 for an overview of clinical trials open for children).

However, if clinical testing cannot be done in a timely fashion, other options must be explored. *In vitro* screening of primary lymphoma cells for binding to therapeutic antibodies and subsequent cytotoxicity can be a first step to choose therapeutic antibodies for individual patients. Another way to assess antibody efficacy may be *in vivo* testing in animal models, such as immunodeficient mice harboring a human immune system (Traggiai et al., 2004). Human B-cell lymphomas can be inoculated into these mice containing an allogeneic, HLA-matched, or a syngenic human immune system. Antibodies can then be administered to generate treatment efficiency data under conditions resembling patient physiology as closely as possible. This experimental setup allows preclinical testing of new antibodies, antibody combinations, or chemoimmunotherapy. It may help to identify new targeted treatment options for children with B-cell lymphoma to make more evidence-based treatment choices for individual patients.

## Conflict of Interest Statement

The authors declare that the research was conducted in the absence of any commercial or financial relationships that could be construed as a potential conflict of interest.
